# Considerations for lactation with Ehlers-Danlos syndrome: a narrative review

**DOI:** 10.1186/s13006-021-00442-9

**Published:** 2022-01-04

**Authors:** Jimi Francis, Darby D. Dickton

**Affiliations:** 1 University of Texas at San Antonio Libraries, San Antonio, TX USA; 2 Foundation for Maternal, Infant, and Lactation Knowledge, San Antonio, TX USA

**Keywords:** Ehlers-Danlos, EDS hypermobility syndrome, Rare disease, Breastfeeding barriers, Breastfeeding support

## Abstract

**Background:**

Ehlers-Danlos syndrome (EDS) is a rare genetic connective tissue condition that is poorly understood in relation to lactation. As diagnostic methods improve, prevalence has increased. EDS, a disorder that impacts connective tissue, is characterized by skin extensibility, joint hypermobility, and fragile tissue which can affect every organ and body system leading to complications during pregnancy, delivery, and the postpartum period. Traits of this disease can cause mild to severe physiologic and functional obstacles during lactation. Unfortunately, there is little clinical evidence and minimal guidance for lactation management, and providers may feel uncomfortable and hesitant to address these concerns with patients due to a lack of readily available resources on the subject and inexperience with such patients. This narrative review describes and discusses the types of EDS, identifying symptoms, considerations, and precautions for care providers to implement during lactation and breastfeeding.

**Methods:**

An electronic search of relevant citations was conducted using the databases Cochrane, PubMed, and Google Scholar from 1 January 2000 to 1 November 2021. Search terms used were Ehlers-Danlos syndrome, Hypermobility Syndrome, breastfeeding, lactation, breastmilk expression, breastmilk collection, human milk expression, human milk collection, and infant feeding. The search of these databases yielded zero results. As no research articles on EDS were directly related to lactation, this narrative review includes articles found that related to the health of mothers relevant to maternal function during lactation.

**Discussion:**

For the healthcare provider, identifying characteristics of EDS can improve the management of lactation challenges. Mothers may experience generalized symptoms from gastrointestinal distress to fatigue or chronic pain, while they also may suffer from more specific joint complaints and injuries, such as dislocations / subluxations, or skin fragility. Such obstacles can generate impediments to breastfeeding and create unique challenges for breastfeeding mothers with EDS.

Unfortunately, new mothers with these symptoms may have them overlooked or not addressed, impacting a mother’s ability to meet her breastfeeding intentions. While there are some published research manuscripts on EDS and pregnancy, there is a lack of information regarding breastfeeding and lactation. Additional research is needed to help guide EDS mothers to achieve their breastfeeding intentions.

## Background

Ehlers-Danlos syndrome (EDS) is a rare genetic connective tissue condition that is poorly understood in relation to lactation but can be characterized by skin extensibility, joint hypermobility, and fragile tissue. These traits can cause physiologic and functional obstacles to breastfeeding for mothers with EDS. Currently, there are 13 recognized subtypes, mainly categorized by key physical determinants that capture the distinctive signs and symptoms of each phenotype. The various EDS manifestations are diagnosed based on the 2017 International Classification-Diagnostic criteria for EDS [1] shown in Table 1. EDS is most often diagnosed by a physician in conjunction with a subsequent referral to a genetic specialist for further evaluation and subcategorization [2]. As determined by a physical evaluation and genetic testing, providers subcategorize these patients in order to accurately identify appropriate risk stratification. Risk stratification helps determine what follow up or imaging is needed over the patient’s lifetime based on the EDS subtype, patient’s age, and symptom onset. Currently, EDS subcategories, many associated with genetic markers, are caused by tissue specific collagen defects usually causing damage to particular systems. For example, there is an Ocular subtype that is defined by the predominant phenotypical expression in the sclera [3], and a Spinal subtype that is defined by extreme vertebral joint laxity causing spinal malalignment [4]. Though most subtypes have been successfully identified genetically [5], Hypermobility EDS seems particularly elusive to genetic linking [2]. As this subtype has not yet been isolated in the genetic code and cannot be diagnosed with genetic testing, it is important to emphasize genetic associations are not required for an Ehlers-Danlos syndrome diagnosis. If no link can be identified, EDS can be diagnosed by phenotypic presentation and at the exclusion of other hypermobility disorders by a physician [6].

**Table 1:** Phenotypic presentation of EDS subtypes pertinent to lactation

EDS Subtype	Protein involved	Genetic basis
Classical	Type V collagen	COL5A1
		COL5A2
	Type I collagen	COL1A1 (Rarely)
Classical-like	Tenascin XB	TNXB
Cardiac-valvular	Type I collagen	COL1A2
Vascular	Type III collagen	COL3A1
Hypermobile	Unknown	Unknown
Arthrochalasia	Type I collagen	COL1A1
		COL1A2
Dermatosparaxis	ADAMTS2	ADAMTS-2
Kyphoscoliotic	PLOD1	LH1
	FKBP14	FKBP22
Brittle cornea syndrome	ZNF469	ZNF469
	PRDM5	PRDM5
Spondylodysplastic	β4GALT7	β4GALT7
	β3GALT6	β3GALT6
	SLC39A13	ZIP13
Musculocontractural	CHST14	D4ST1
	DSE	DSE
Myopathic	COL12A1	Type XII collagen
Periodontal	C1R	C1r

Foundationally, EDS causes dysfunction in collagen fibers and while subtypes describe specific body systems or mechanisms that are more directly affected [7], all patients with EDS, to some extent, experience generalized, broad systemic complaints. Tissue laxity (often identified as “stretchy skin”), joint hypermobility (frequently identified as “loose / unstable joints”), difficulties with proprioception, or acute awareness of where body parts are in space, and other generalized systemic problems such as intestinal dysmotility, impaired healing, and inappropriate scarring can be present [8]. Due to the proprioception abnormalities, patients with EDS are frequently unaware their joints are sliding out of alignment or into a dangerously hyper-flexed or hyper-extended state until they experience pain. On a cellular level, in normal individuals, connective tissue provides strength, structure, and elasticity to body systems allowing movement within normal limits but not beyond those limits [9]. Connective tissue in the EDS mother can and will allow for unintentional movement beyond normal limits ranging from mild examples such as ligament strain from hyper-extension to more severe manifestations such as dislocations, causing chronic forms of damage, pain, and dysfunction [10].

While it was once thought to be an extraordinarily rare condition, the prevalence of EDS using newer estimation models could be as common as 194 per 100,000 in 2016 / 2017 [11]. As genetic research has increased greatly in this area, the understanding of EDS subtypes and variations has become an expanding field of study [8]. There can be an overlap of signs and symptoms with other connective tissue disorders and, challengingly, some current definitions for clinical criteria are non-specific. Given the infrequency of provider-patient interactions for this disease, even fewer providers feel comfortable making the necessary recommendations [12] or adjustments to improve the mother-baby experience of new mothers coping with EDS.

As EDS is a disorder that impacts connective tissue, every organ and body system can be affected by poor structural integrity, and when combined with the major physiologic and physical changes of pregnancy due to the hormone relaxin, a pleiotropic hormone [13], complications are common during pregnancy and at delivery, including uterine rupture, precipitous delivery (< 4 h), arterial dissection / rupture, and miscarriage [14, 15]. These complications can create impediments to lactation, whether feeding a baby at the breast or expressing milk. Other unique challenges arise given the weight of mammary tissue during lactation, especially during secretory activation, with the increased laxity of Cooper’s ligaments, which suspend breast tissue. Pain caused by the stretching of the ligaments can make the positioning and the ergonomics of feeding at the breast challenging, as well as potentially interfering with and / or inhibiting milk production or flow. Unfortunately, a lack of readily available resources on the subject [16] paired with little clinical evidence means there is minimal guidance for optimal lactation management of EDS patients, making many providers hesitant to offer care recommendations [17]. Based on the authors’ experience this can make a diagnosis of EDS challenging and often translates into general apprehension for clinicians managing mothers with EDS.

The purpose of this narrative review is to describe and synthesize the available information that pertains to caring for lactating mothers with EDS. Increasing the understanding of the syndrome and the impact various subtypes may pose for lactation is crucial for better management and support of those new mothers coping with EDS while lactating, whether feeding at the breast and / or expressing their milk. While this review focuses on EDS, the following recommendations are based on symptomatic presentations that could be applied to other similar systemic diseases.

## Methods

An electronic search of relevant citations was conducted using databases Cochrane, PubMed, and Google Scholar. The keyword search terms were Ehlers-Danlos syndrome, Hypermobility Syndrome, breastfeeding, lactation, breastmilk expression, breastmilk collection, human milk expression, human milk collection, and infant feeding. The dates for this search were from 1 January 2000 to 1 November 2021. The search of Cochrane, PubMed, and Google Scholar yielded zero results. As no research articles on EDS were directly related to lactation, this narrative review includes articles found that related to the health of mothers relevant to maternal function during lactation. This narrative review includes information based on general and specific complaints associated with EDS which are pertinent to lactation.

## Discussion

Mothers with EDS may be frustrated by providers unable to diagnose their reports of pain or system-wide disturbances [12]. Identifying characteristic of EDS and providing supportive care can improve the management of lactation challenges. For the ease of reference, the topics have been organized by relevant body system. Specific complaints or challenges are addressed under these body systems headings. Strategies pertinent to EDS management during lactation are shown in Table 2 [1, 3, 4, 6, 9, 14].

**Table 2:** Strategies pertinent to EDS management during lactation

EDS Subtype	Sign-symptoms	Suggestions for breastfeeding support		
		During pregnancy	Initiating breastfeeding	Throughout lactation
Classical	*Skin can be easily torn and will not repair itself well or quickly *Joint hypermobility *Atrophic scarring, poor healing *Skin hyper-extensibility	Providing breastfeeding education early in the third trimester can be helpful as preterm labor can occur	Prevent nipple trauma through early evaluation of latch	Prevent scarring and manage wounds with on-going feeding assessment
		Splinting the pelvis, ligaments, and joints can help with pelvic pain	Assess positioning during breastfeeding to prevent injury	Frequent feedings and slow weaning minimize engorgement
Classical-like	*Soft-velvety skin (without the typical atrophic scarring seen in classical EDS) leads to easy irritation.	Be conscious of rough fabrics and materials including silicone which can stick to the skin and must be removed gently to avoid tearing of the skin	Ensure optimized latch. Gentle breast massage may be useful for alleviating discomfort of secretory activation	Avoid positions and equipment that shear or create torsion of the tissue
Cardiac-valvular	Progressive cardiac and valve problems Dizziness and fainting can occur	Will likely require regular follow-up with Cardiology Practice slow standing	Will likely require follow-up with Cardiology Report palpitations and new symptoms	Reminders to pick the infant up after standing can help prevent falls
Vascular	Unusual bruising for no apparent cause Postpartum hemorrhage can occur	Monitor for orthostatic difficulties, and practice safe habits when first standing; avoid rushed movements	Placing the infant skin-to-skin within the first hour of life is crucial	Reminders to pick the infant up after standing can help prevent falls
Hypermobile	Severe generalized joint hypermobility. Separation of the pubic symphysis and coccyx dislocation have been reported	Prevent injuries using focused and slow movements	Physical support and positioning modifications may be needed for basic infant care	Monitor for increasing pain difficulties, check safety of medication regimen for breastfeeding
Arthrochalasia	Multiple dislocations and / or subluxations	Prepare to practice aggressive, daily splinting measures to support joints	Exercise caution with movements and monitor for tissue trauma	Can complicate use of a breast pump
Dermatosparaxis	Extreme skin fragility and severe susceptibility of bruising	Requires a specific focus on skin care and precautions	Consider use of skin barrier protectant if using a pump	Monitor for chronic wound development
Kyphoscoliotic	Dislocations and / or subluxations of the shoulders, hips and knees	Abdominal bracing may be beneficial during the third trimester	For hearing loss may need visual alerts to signal infant needs	Special considerations should be given to ergonomics when seated
Brittle cornea syndrome	Practice classic considerations	For new symptoms, seek medical eye care	Use touch to increase awareness of baby’s positioning if difficulty seeing	Identify strategies to assist in infant care that are touch or sound focused
Spondylodysplastic	Reduced muscle tone and rigidity can occur	Refer to physical therapist; stretching needs to be carefully balanced with strengthening	Match comfort with good ergonomic positioning to prevent injuries	Watch for good ergonomic positioning to improve long term outcomes
Musculocontractural	Risk for hematomas	Practice skin and tissue considerations An exercise ball may be helpful	Use support devices to use burden on musculature	Rehabilitative tape may be useful to stabilize ligaments during breastfeeding sessions
Myopathic	Muscle weakness	Use of a pelvic belt may be useful	Mother may need additional structural and positioning support during lactation	Monitor for chronic symptom development or worsening
Periodontal	Inflammation of the tissue around teeth	Can lead to food avoidance. Refer to a dietitian / nutritionist to ensure adequate food intake	Follow-up with a dietitian / nutritionist to reassess nutritional needs during lactation	Continued follow-up with a dietitian / nutritionist to protect milk production

*These traits will likely be seen in most EDS variations and as such these precautions should be considered in all EDS mothers

### Joints and muscles

In patients with EDS, dislocations and / or subluxations can occur with even the slightest pressure. For mothers with the syndrome, positioning a baby at the breast or supporting a breast heavy with milk can provoke such injuries. Routine tasks like hair washing may lead to shoulder dislocations with the breast weight changes associated with secretory activation in the early postpartum period. Even tasks which shift the weight of the breast, such as putting down the flap on a bra, or lack of support when latching the infant, can cause internal stress in the breast tissue [18]. On a microscopic level, subtle shifts of the increased mass of breast tissue can cause shearing injuries to the smallest areas of the circummammary ligaments [19] dispersed throughout the breast as superficial tissue moves in one direction while the deeper tissue moves in a different direction [20]. Educating mothers on multiple positions for breastfeeding and assisting them with guided practice during pregnancy can help mothers with EDS build confidence as well as identifying problem areas that may need bracing, such as wrists or fingers. The lactation specialist can help them create adequate physical support using pillows or blanket rolls to minimize strains and prevent injuries based on the mother’s needs. Techniques to reinforce appropriate posture and ergonomics can also address the difficulties for mothers who suffer joint strain due to lack of body awareness caused by the known proprioceptive abnormalities that may present in EDS [9, 21]. Preparation for infant care and practicing these tasks during pregnancy with slow, controlled strength conditioning can reduce injury risk with simple routine tasks or repeated motions, like putting on a bra, or picking up their infant, which can cause varying degrees of injury to the ligaments of the shoulder [22]. Reminding patients, who struggle with suspected shearing injuries, to support the breast while bringing the infant to the breast rather than bringing the breast to the infant can be particularly prudent to decrease the risk of circummammary ligament damage in mothers with EDS. While wearing a supportive bra, joint braces, proprioceptive garments, using physical supports, and regular, specific physical strengthening can decrease the risk of injury, it is important to plan for pain management [23] of injuries so that these mothers can be empowered with skills to optimize their situation. Establishing relationships with other care providers to create a health care team that includes ideally a physiotherapist and a nutritionist is important for the total care of these patients.

It is also important to note that EDS patients will often find positions such as tucking in their knees or crossing their legs provide temporary relief for muscle fatigue. These positions do slowly stretch out ligaments and tendons due to uncontrolled hyper-flexing during this muscle relaxation and can be damaging long-term if sustained for an extended period. Patients will often be unaware and should be educated and frequently reminded of these challenges to best initiate ergonomic posture strategies, particularly during feeding sessions. If left unchecked tucked in knees and crossed legs can cause further pain and joint instability.

### Pain

EDS patients can have chronic pain, often from irritated joints and slow healing of injuries. This chronic pain requires appropriate pain interventions that serve to decrease pain intensity [24, 25]. Many women with EDS experience neuropathic pain and / or small fiber neuropathy [26]. Their connective tissue can be hyper-elastic without resilience and fragile, but the nerves are not hyper-elastic. This may be a cause of pain, particularly if latch is poor or the infant is pulled from the breast without releasing the latch pressure. Raynaud’s syndrome is also very common in EDS [27, 28] and can contribute to nipple and breast pain. Often, without awareness, EDS patients develop a fidget or erratic frequency to their movements to accommodate small muscle and nerve firings while protecting themselves from isolated muscle failure secondary to fatigue. It is common for EDS patients to have peripartum musculoskeletal [15] and visceral pain [29] which can be exacerbated by the surge of relaxin (which promotes connective tissue remodeling via increased collagen turnover) [30], essential for the ligamentous laxity necessary for pelvic flexibility to accommodate fetal development and vaginal delivery. While the surge peaks in the first trimester, it slowly decreases throughout the second trimester and maintains a stable level until parturition where it drops precipitously [31].

Although the relaxin level drops, the ligamentous laxity can persist into the postpartum period, compounding EDS symptoms, manifesting as pelvic floor distension creating pain or pelvic girdle discomfort. The weight of a lactating breast combined with ligament laxity can create a burning sensation deep in the breast (as reported to the authors in clinical practice). Warm compresses [32] and magnesium supplementation [33] have been reported to be helpful for alleviating symptoms. Nifedipine has been reported to successfully manage nipple vasospasms [34].

Use of a stability ball seat has been reported to the authors by EDS moms to give them a way to express the EDS muscle fidget and use a variety of muscle groups to prevent the tiring of singular muscles. Rhythmic sensory stimulation [35], transcutaneous electrical nerve stimulation (TENS) [36], lidocaine patches [37], warming pads / compresses [38], and joint injections [39] may also be useful techniques to manage pain. Electronic methods and alternative remedies may need to be assessed in depth to ensure the mother is being informed fully as to the lactation considerations and risks while using these interventions.

**Fig. 1:**
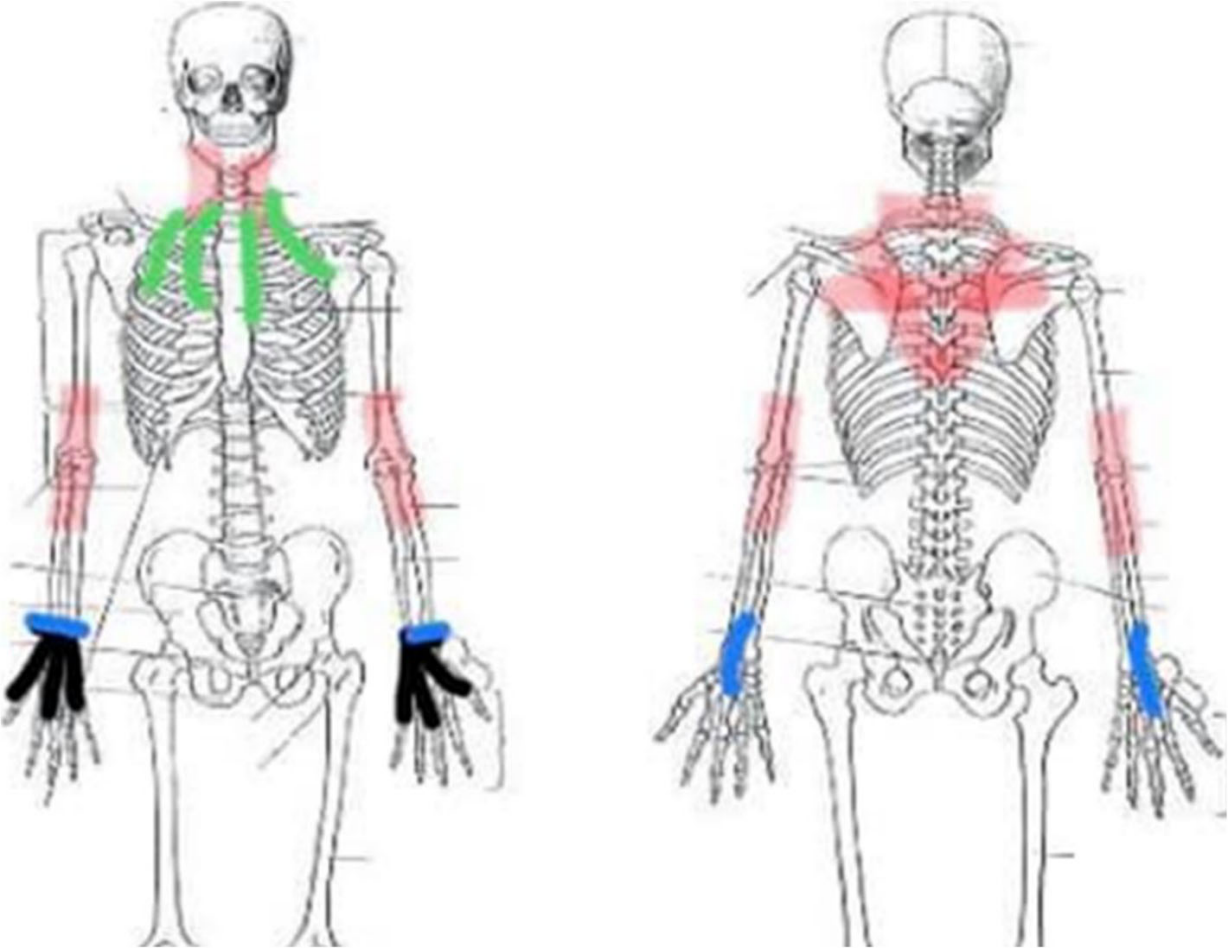
Rehabilitative tape positioning

Use of subtle bouncing on an exercise ball has also been reported to ease pelvic and low back discomfort during pregnancy, labor, and postnatally for non-EDS patients [40–42] and may be effective for EDS patients as reports to the authors indicate. Structurally speaking, splinting the pelvis, ligaments, and joints can help with pelvic pain. Sacro-iliac and uterine splinting can be particularly helpful in alleviating anterior and posterior pelvic discomfort. Pelvic discomfort can continue after the baby’s birth and may require physiotherapy as well as analgesia for pain management [43]. Assisting new mothers to find adaptive measures for discomfort including supportive clothing and / or devices such as braces can minimize or prevent discomfort. Gentle breast massage [44] along with bracing and support garments was found to be useful for alleviating the pain manifesting from these various musculoskeletal micro-injuries. However, the use of rehabilitative tape, designed originally as a ligamentous stabilizing adhesive [45] which creates physiologically appropriate tension to externally splint the body [46] and other therapeutic non-invasive joint support modalities, have been increasingly researched over the last several years [47]. Such principles can be applied well to the unique circumstance mothers with EDS encounter. However, it must be noted that rehabilitative tape should be used cautiously in mothers who have moderate to severe dermal manifestations of EDS. In mild cases, skin barrier film may help but the mother should be monitored to ensure a successful positive response. A diagram of possible tape positioning is shown in Fig. 1, provided in a personal communication by Bryna Sampey of Doula My Soul, IBCLC [48].

### Fatigue

For EDS patients, pain is often associated with fatigue, which can be compounded by sleep deprivation that often accompanies the arrival of a neonate. Helping a new EDS mother with finding adequate resources to manage fatigue is especially important. These resources can include neighbors, friends, church members, and family who all want to help make life easier for the new mother as well as providing the additional assistance needed to promote successful lactation. Equally important is giving the mother with EDS a way to explain her condition and circumstances to those whom she chooses, so she might feel better understood. Encouraging her to reach out to her support network and accepting their “help”, as they are often delighted to provide meals, occasional cleaning, or to deliver groceries as a way of allowing help with the new family that will leave the new mother more time to focus on managing her condition while acclimating to life with a new infant.

### Gastrointestinal

Gastrointestinal problems include constipation, diarrhea, and / or reflux [49]. As people with EDS often experience nausea and abdominal pain that may decrease their desire to eat, adequate maternal nutrient intake can be a challenge [50]. If mothers with EDS experience diarrhea, it is essential that they stay hydrated for maternal wellbeing. Suggesting mothers place a glass of water on a table or nightstand where they will be feeding their infant at the breast can help them remember to maintain adequate hydration. Gastrointestinal effects are often dismissed as “stress-related” stomach issues and can be difficult to separate from stress related somatization [50, 51] It is also important for mothers suffering the gastrointestinal aspects of EDS to be monitored for malnutrition and weight maintenance during lactation. Individuals with gastrointestinal symptoms may benefit from advice from a nutritionist.

### Skin and fascial tissues

The skin of the EDS mother can be fragile [52], bruising or tearing easily with incorrect latch, infant pulling on the nipple to maintain latch, or an infant biting. Preventive and remedial options are an important aspect of giving mothers the power of self-care in their situation. Engorgement in mothers with hyperplastic breast tissue can be very extreme as their tissue will continue expanding with the increasing pressure if milk is not removed. In theory, some mothers may struggle with achieving let-down due to difficulties with proprioceptive nerves [18]. Conversely, the authors have observed that some mothers with EDS have a strong let-down with high pressure flow of the milk due to the dysfunctional collagen which coordinates the smooth muscle movement of mammary tissue. Engorgement is worth preventing with regular, frequent feeds in the early days of lactation. Extreme engorgement can also occur during breastfeeding transitions, such as infant growth spurts and cessation of breastfeeding, making education on transitioning and gradual weaning techniques an imperative.

In cases of let-down difficulty, stress reduction, reassurance, and breast massage can help provide stronger visual / auditory / tactile cues to stimulate oxytocin release and myoepithelial contraction of the alveoli and milk ducts. For those with strong let-down, repositioning the infant into a sitting position can help the infant manage the high-pressure flow that results from strong let-down.

### Dysautonomia

Dysautonomia is a dysfunction of the nerves that regulate involuntary body functions, such as perspiration, blood pressure, and heart rate. Many patients with EDS have associated diagnoses that classify as dysautonomia such as postural orthostatic tachycardia syndrome (POTS) [53, 54]. These problems with the autonomic (“fight, flight, or freeze”) nervous system can lead to rapid lowering of blood pressure often upon standing, or an excessively fast heart rate, which can be challenging to cope with as such symptoms, like dizziness, palpitations, or (near) fainting, are often not quantifiable and can be misdiagnosed as the “anxiety” of being a new mother. While these autonomic symptoms may require medication, there are often mechanical and precautionary strategies that are also implemented, such as compression leggings and particular physiotherapy regimens. Mothers who experience near fainting with standing due to dysautonomia should be cautioned against standing quickly and should be advised, for the safety of both, to pick the infant up after standing rather than hold the infant and stand up.

## Conclusions

Although scant information is available on mothers with EDS and their challenges during lactation, from the research available and the commonly accepted aspects of EDS treatment recommendations can be compiled. Given that this topic is relatively novel and modern medicine is working towards improving our understanding of EDS there is a limitation to the application of generalized recommendations. These compiled discussions of treatment options should be used at the discretion and best judgment of the necessary medical providers.

Each mother may have a combination of signs and symptoms that are unique to them, and all mothers should be treated on a case-by-case basis. Some mothers with EDS may have various types of pain with feeding at the breast or expressing milk while others may have none. As with any patient, it is crucial to take a full health history to develop a precise, targeted care plan. As EDS has 13 subtypes, there can be overlaps in the signs and symptoms of a particular patient to several subtypes, and while every subtype of EDS has unique risks for mothers [55], there is a great deal of overlap in terms of the experiences of such mothers in their obstacles to a successful lactation experience. Unfortunately, their symptoms may be overlooked or not addressed given the lack of practitioners’ exposure to the condition. Watching a mother with EDS, listening closely to what is said, and working with the health care team to provide all aspects of lactation support are crucial to helping this unique patient on the path to achieving their lactation goals. Research is needed to address gaps in existing knowledge regarding EDS and lactation which will facilitate evidence-based practice in the support of lactating mothers with EDS.

## Data Availability

Not applicable.

## Abbreviations

EDS: Ehlers-Danlos syndrome
GI: Gastrointestinal
KT tape: Kinesiology therapeutic tape
TENS: Transcutaneous electrical nerve stimulation
